# The NLS-Based Nonlinear Grey Multivariate Model for Forecasting Pollutant Emissions in China

**DOI:** 10.3390/ijerph15030471

**Published:** 2018-03-08

**Authors:** Ling-Ling Pei, Qin Li, Zheng-Xin Wang

**Affiliations:** 1 School of Business Administration, Zhejiang University of Finance & Economics, Hangzhou 310018, China; linglingpei@zufe.edu.cn; 2 School of Economics, Zhejiang University of Finance & Economics, Hangzhou 310018, China; zxwang@zufe.edu.cn

**Keywords:** pollutant discharge, economic growth, NLS method, GM (1, *N*) model, TNGM (1, *N*) model

## Abstract

The relationship between pollutant discharge and economic growth has been a major research focus in environmental economics. To accurately estimate the nonlinear change law of China’s pollutant discharge with economic growth, this study establishes a transformed nonlinear grey multivariable (TNGM (1, *N*)) model based on the nonlinear least square (NLS) method. The Gauss–Seidel iterative algorithm was used to solve the parameters of the TNGM (1, *N*) model based on the NLS basic principle. This algorithm improves the precision of the model by continuous iteration and constantly approximating the optimal regression coefficient of the nonlinear model. In our empirical analysis, the traditional grey multivariate model GM (1, *N*) and the NLS-based TNGM (1, *N*) models were respectively adopted to forecast and analyze the relationship among wastewater discharge per capita (WDPC), and per capita emissions of SO_2_ and dust, alongside GDP per capita in China during the period 1996–2015. Results indicated that the NLS algorithm is able to effectively help the grey multivariable model identify the nonlinear relationship between pollutant discharge and economic growth. The results show that the NLS-based TNGM (1, *N*) model presents greater precision when forecasting WDPC, SO_2_ emissions and dust emissions per capita, compared to the traditional GM (1, *N*) model; WDPC indicates a growing tendency aligned with the growth of GDP, while the per capita emissions of SO_2_ and dust reduce accordingly.

## 1. Introduction

China has witnessed rapid economic development, acknowledged globally since political reform and opening-up of trade and communications in the last three decades. China’s GDP in 2016 was 201.5 times greater than that of 1978, when calculation is based on a constant price, which was the second largest following the USA. However, such growth leads to severe issues related to environmental pollution. According to the China National Environmental Protection Plan, in the 13th Five-Year Plan issued by the State Council of China, China has seen heavy environmental pollution and large areas containing great amounts of pollutant discharge. Data obtained until 2016 showed that the discharge of the pollutants including chemical oxygen demand (COD) and sulfur dioxide had reached approximately 20 million tons. The air quality of the cities, of which the environmental carrying capacity exceeded or approached upper limit (78.4%), failed to reach the environmental standard, and according to the public survey, days with severe or even worse pollution degree accounted for 3.2% of the whole year. Environmental pollution has been seriously affecting our daily lives [1,2] and is expected to restrain economic development [3]. In recent years, the relationship between economic development and pollutant discharge has been widely researched. As China now confronts both issues—slow economic growth and decreasing environmental quality—it is significant for China to realize its economic transition in The Thirteenth Five-Year Plan, by exploring the relationship between economic growth and pollutant discharge (two variables) as well as their changing trends.

The rest of this paper is organized as follows: Section 2 is the literature review which introduces and reviews current literature concerning the relationship between pollution discharge and economic growth, forecasted using the grey multivariable models (GM (1, *N*)). Section 3 introduces the GM (1, *N*) model, the transformed nonlinear grey multivariable (TNGM) (1, *N*) and the parameter estimation using nonlinear least square (NLS)-based TNGM (1, *N*) (NLS-TNGM (1, *N*)). Section 4 illustrates the empirical research of the relationship between the emissions of the three pollutants (wastewater discharge per capita (WDPC), SO_2_ and dust) and the GDP per capita in China using the GM (1, *N*) and NLS-TNGM (1, *N*), and compares the empirical results of the two models. Section 5 presents a conclusion.

## 2. Literature Review

### 2.1. Literature Concerning the Relationship between Pollutants and Economic Growth

The relationship between pollutant emissions and economic growth has been a major research subject in energy economics. Most existing studies focus on the hypothesis on whether the environmental Kuznets curve (EKC) exists or not. Grossman and Krueger—two economists from the USA—verified the relationship between environment quality and per capita income for the first time, and they confirmed that the relationship between environmental quality and economic growth agrees with the inverted U-shaped correlation of Kuznets curves. Subsequently, many studies carried out empirical validations on the existence of the EKC; unfortunately, they failed to obtain consistent conclusions. Furthermore, some research validated the existence of the inverted U-shaped EKC [4]; however, other studies verified the absence of the EKC [5,6,7,8]. Moreover, some scholars verified that there is an N shape or an inverted N-shape EKC curve [9,10,11,12]. As a matter of fact, whether the EKC is verified to exist or not, the varying shapes of the EKC curves are largely dependent on the selection of pollution indexes and research approaches. Some scholars have utilized different methods to research the relationship between the emissions of pollutants including SO_2_, dust and NOx and economic growth, for instance, with linear quadratic and cubic regressions [13,14,15], the panel data regression model [16,17], the Panel Smooth Transition Regression [18], etc. However, most of the studies explored the linear relationship between pollutant discharge and economic growth based on large-scale samples, with little attention paid to the nonlinear relationship between the emissions of pollutants and economic growth.

### 2.2. Research Progress of the GM(1, N) Model

Grey system theory was proposed by Prof. Deng Julong in 1982 [19]. Grey system model forecasting is generated by accumulation to generate randomness in a weakening series, and to determine the relationships governing changes in a system. The simulation and accuracy of the grey model would be higher when modeling based on qualitative analysis. In recent years, it has been an indispensable tool for academic research and solving practical problems and has been widely applied in the energy field [20,21,22]. The improvement of grey model mainly focuses on improving the generation of sequences [23], the background value [24,25], optimizing the initial condition [21] and time response formula [26], and improving the method of parameter estimation [27]. The GM (1, *N*) forecasting model, as an important branch of grey forecasting theory, can preferably reflect the relationships (mutual influences and restrictions) among different variables in the system by researching the relationship between sequence features of time series and driving factors of the system. The GM (1, *N*) has gradually become a research focus of grey theory predication as it is more consistent with real development law of the grey system. Existing GM (1, *N*) studies mainly include the following: (1) Improving the structure of the GM (1, *N*) model: Tien [28] found that the solution for the whitening differential equation of the traditional GM (1, *N*) model exhibits a greater error and used convolution integral to improve the solving approach to further propose a novel grey prediction model with convolution integral GMC (1, *N*). Moreover, Tien verified the correctness of the novel solving method of the GMC (1, *N*) model. Afterwards, GMC (1, *N*) was modified and applied in different fields, achieving favorable performance [29,30,31]. To satisfy different application demands, Tien put forward three modified models—the deterministic grey dynamic model with convolution integral-DGDMC (1, *N*) [32], the interval grey dynamic model by convolution integral-IGDMC (1, *N*) [33] and the First-pair-of-data based on GMC (1, *N*)-FGMC (1, *N*) [34]—Zeng et al. [35] introduced a linear correction item and grey action quantity into GM (1, *N*) to improve its structural defects, and then the improved model was applied to forecast the amount of Beijing’s motor vehicles, the effectiveness of which was validated. (2) The optimization of parameters for GM (1, *N*): Hsu [29] and Pei et al. [36] used genetic algorithms to optimize the interpolated coefficients of the background values in GM (1, *N*), and then the optimized predication model was employed to forecast the Taiwanese-integrated circuit output and the input–output system of China. Deng Julong conducted a detailed validation of different aspects [37,38]. Ding et al. [39] proposed an optimized grey model by modifying the background value to predict the trends of the driving variables, and optimize the adjustment coefficient in the new model, the efficacy of which was verified by predicting the Chinese CO_2_ emissions from fuel combustion. Guo et al. [40] proposed a novel comprehensive adaptive grey model (CAGM) (1, *N*) with three improvements. (3) The issues regarding time delay of the GM (1, *N*) model: Hao et al. [41] improved the forecasting precision of the GM (1, *N*) model by determining time-delay parameters of driving factors using grey correlation analysis; Mao et al. [42] extended time-delay GM (1, *N*) into a fractional GM (1, *N*) model by introducing a fractional-order accumulation operator. Furthermore, they used particle swarm optimization (PSO) to determine the orders generated by the optimal fractional order accumulation of the model. Their research expanded the application range of the GM (1, *N*) model; Wang [43] demonstrated a derived time-delay GM (1, *N*) model by using the time-delay coefficients of driving factors to reflect the influences of the data at different time series on the system behavior sequence of the system; Dang et al. [44] introduced the regulation coefficient and effect coefficient for controlling items of driving factors to build a new discrete time-delay multivariate grey model (DTMGM (1, *N*). They adopted a fractal dimension extension identification method based on a grey system to identify the regulation coefficient and determine the time-delay parameters and different driving factors; furthermore, PSO was used to solve the effect coefficient using PSO so as to reflect the influences of driving factors for data at a previous period on the system behavior sequence for the data at the current period. Lastly, they validated the effectiveness of the proposed model by taking energy consumption data of Jiangsu Province in China as an example. These studies increased the adaptability and fitness of the proposed grey models to a certain degree; however, they neglected the nonlinear features of the models. The exponent at the right side of the grey differential equation of GM (1, *N*) can present the nonlinear relationship of the system behavior sequence. Ma and Liu et al. [45] proposed the kernel-based GM (1, *N*) model to use kernel functions to estimate the unknown function of the model. By doing so, the nonlinear relationship between input and output series can be described. Additionally, they made an empirical comparison between existing linear multivariate grey models and the LSSVM with an aim to improve the forecasting precision of the kernel-based GM (1, *N*) model. Wang [46] introduced nonlinear parameters into the GMC (1, *N*) model to establish a novel NGMC (1, *N*) which can preferably reflect the causal relationship between input and output variables. They applied this model to reveal the relationship between China’s economic output and SO_2_ emission, validating the effectiveness of the proposed model. Meanwhile, with an aim of minimizing the mean error of the model and relationship between the parameters of the model as constraints, solving unknown parameters of the model by using two constructed nonlinear programming models can also promote the forecasting precision of the model [47].

As a matter of fact, the relationship between pollutant discharge and economic development is usually shown to be nonlinear instead of linear. Therefore, econometrics models adopted by most scholars are only able to describe linear problems and cannot be used in research of nonlinear relationships. Given that the least square method (LSM) is inapplicable to the solution of nonlinear problems, this research employed the LSM-based TNGM (1, *N*) to estimate the parameters of the model and used the Gauss–Seidel iteration approach to continuously approximate the optimal regression coefficient of the nonlinear model so as to improve the forecasting precision of the model. Lastly, NLS-TNGM (1, *N*) was utilized to research the relationship between the per capita emissions of pollutants (WDPC, SO_2_ and dust) and GDP per capita in China. This study is expected to provide the Chinese government with decision-making references for environmental control and improvement, as well as energy saving and emissions reduction.

## 3. Methodology

### 3.1. Theoretical Hypotheses

Since China’s reform and opening up, the country’s economy has developed rapidly, but it simultaneously led to a sharp decline in environmental quality—all kinds of pollutant emissions are increasing. The government has formulated relevant policies, but the intensity of remediation is different in relation to different pollutants. Therefore, for the study of the relationship between pollutants and economic growth, this paper proposes the following three assumptions:

**Hypothesis** **1.** There is a monotonically increasing relationship between WDPC and GDP per capita.

**Hypothesis** **2.** There is a decreasing relationship between SO_2_ emissions per capita and GDP per capita.

**Hypothesis** **3.** There is a relationship between dust emissions per capita and GDP per capita firstly decreasing and then increasing.

### 3.2. Grey Multivariable Models (GM (1, N))

The GM (1, *N*) model is a first-order multivariate grey model which contains a system behavior variable and N-1 influencing variables; the model can analyze the effect of multiple influencing variables on system behavior. The system behavior variables can be predicted in the case of knowing the changing trend of influencing variables. The specific modeling process is as follows:

**Definition** **1.** $X_{1}^{\left(0\right)}=\left(x_{1}^{\left(0\right)}\left(1\right),x_{1}^{\left(0\right)}\left(2\right),\cdots ,x_{1}^{\left(0\right)}\left(n\right)\right)$ *is assumed to be the data series of system features, while*

$$\begin{array}{c} X_{2}^{\left(0\right)}=\left(x_{2}^{\left(0\right)}(1),x_{2}^{\left(0\right)}(2),\cdots ,x_{2}^{\left(0\right)}(n)\right)\\ X_{3}^{\left(0\right)}=\left(x_{3}^{\left(0\right)}(1),x_{3}^{\left(0\right)}(2),\cdots ,x_{3}^{\left(0\right)}(n)\right)\\ \vdots \\ X_{N}^{\left(0\right)}=\left(x_{N}^{\left(0\right)}(1),x_{N}^{\left(0\right)}(2),\cdots ,x_{N}^{\left(0\right)}(n)\right) \end{array},$$

They are the data series of relevant factors. $X_{i}^{\left(1\right)}$ is the series (i=2,3,⋯,N) generated by first-order accumulation (1-AGO), while $Z_{1}^{\left(1\right)}$ represents the data series for the nearest-neighbor mean of $X_{1}^{\left(1\right)}$ and is expressed as

(1) $$x_{1}^{\left(0\right)}(k)+az_{1}^{\left(1\right)}(k)=\sum_{i=2}^{N}b_{i}x_{i}^{\left(1\right)}(k)$$

which is the grey multivariable model, and abbreviated as GM (1, *N*) [48,49].

**Definition** **2.** *In the GM (1, N) model,* −a *is the system development coefficient,* $b_{i}x_{i}^{\left(1\right)}(k)$ *refers to driving items,* $b_{i}$ *represents the driving coefficient and* $\hat{\alpha }={\left[a,b_{2},\ldots ,b_{N}\right]}^{T}$ *is the series of parameters.*

**Theorem** **1.** *Supposing* $X_{1}^{\left(0\right)}=\left(x_{1}^{\left(0\right)}(1),x_{1}^{\left(0\right)}(2),\cdots ,x_{1}^{\left(0\right)}(n)\right)$ *is the data series of system features,* $X_{i}^{\left(0\right)}\left(i=2,3,\ldots ,N\right)$ *is the data series of relevant factors, while* $X_{i}^{\left(1\right)}$ *is the 1-AGO series of* $X_{i}^{\left(0\right)}$ *and* $Z_{1}^{\left(1\right)}$ *is the data series of nearest-neighbor means of* $X_{1}^{\left(1\right)}$*.*

The series of parameters $\hat{\alpha }={\left[a,b_{2},\ldots ,b_{N}\right]}^{T}$ can be solved by the least square method (LSM) [50] and written as:

(2) $$\hat{\alpha }={\left[a,b_{2},\ldots ,b_{N}\right]}^{T}={\left(B^{T}B\right)}^{-1}B^{T}Y$$

where

$$B=\left[\begin{array}{cccc} -z_{1}^{\left(1\right)}\left(2\right) & x_{2}^{\left(1\right)}\left(2\right) & \cdots & x_{N}^{\left(1\right)}\left(2\right)\\ -z_{1}^{\left(1\right)}\left(3\right) & x_{2}^{\left(1\right)}\left(3\right) & \cdots & x_{N}^{\left(1\right)}\left(3\right)\\ \vdots & \vdots & \vdots & \vdots \\ -z_{1}^{\left(1\right)}\left(n\right) & x_{2}^{\left(1\right)}\left(n\right) & \cdots & x_{N}^{\left(1\right)}\left(n\right) \end{array}\right]Y=\left[\begin{array}{c} x_{1}^{\left(0\right)}\left(2\right)\\ x_{1}^{\left(0\right)}\left(3\right)\\ \vdots \\ x_{1}^{\left(0\right)}\left(n\right) \end{array}\right]$$

**Definition** **3.** *Supposing* $\hat{\alpha }={\left[a,b_{2},\ldots ,b_{N}\right]}^{T}$*, we obtain*

(3) $$\frac{dx_{1}^{\left(1\right)}}{dt}+ax_{1}^{\left(1\right)}=b_{2}x_{2}^{\left(1\right)}+b_{3}x_{3}^{\left(1\right)}+\ldots +b_{N}x_{N}^{\left(1\right)}$$

*which is the whitening differential equation of the traditional GM (1, N) model.*

**Theorem** **2.** *Supposing* $X_{i}^{\left(0\right)}$ *and* $X_{i}^{\left(1\right)}\left(i=2,3,\ldots ,N\right)$*,* B,Y *are described as Theorem 1.*

(4) $$\hat{\alpha }={\left[a,b_{2},\ldots ,b_{N}\right]}^{T}={\left(B^{T}B\right)}^{-1}B^{T}Y$$

The solution of the whitening differential equation $\frac{dx_{1}^{\left(1\right)}}{dt}+ax_{1}^{\left(1\right)}=\sum_{i=2}^{N}b_{i}x_{i}^{\left(1\right)}$ is expressed as

(5) $$\begin{array}{cc} x_{1}^{\left(1\right)}\left(t\right) & =e^{-at}\left\{\sum_{i=2}^{N}\int b_{i}\left(x_{i}^{\left(1\right)}\left(t\right)\right)e^{at}dt+x_{{}^{1}}^{\left(1\right)}\left(0\right)-\sum_{i=2}^{N}\int b_{i}\left(x_{i}^{\left(1\right)}\left(0\right)\right)dt\right\}\\ & =e^{-at}\left\{x_{{}^{1}}^{\left(1\right)}\left(0\right)-t\sum_{i=2}^{N}b_{i}\left(x_{i}^{\left(1\right)}\left(0\right)\right)+\sum_{i=2}^{N}\int b_{i}\left(x_{i}^{\left(1\right)}\left(t\right)\right)e^{at}dt\right\} \end{array}$$

when $X_{i}^{\left(1\right)}\left(i=2,3,\ldots ,N\right)$ changes slightly, $\sum_{i=2}^{N}b_{i}\left(x_{i}^{\left(1\right)}\left(k\right)\right)$ is seen as a grey constant, while the approximate time response of $x_{1}^{\left(0\right)}(k)+az_{1}^{\left(1\right)}(k)=\sum_{i=2}^{N}b_{i}x_{i}^{\left(1\right)}(k)$ is written as [51].

(6) $${\hat{x}}_{1}^{\left(1\right)}\left(k+1\right)=\left[x_{1}^{\left(1\right)}\left(0\right)-\frac{1}{a}\sum_{i=2}^{N}b_{i}\left(x_{i}^{\left(1\right)}\left(k+1\right)\right)\right]e^{-ak}+\frac{1}{a}\sum_{i=2}^{N}b_{i}\left(x_{i}^{\left(1\right)}\left(k+1\right)\right)$$

where $x_{1}^{\left(1\right)}\left(0\right)$ is set to be $x_{1}^{\left(1\right)}\left(1\right)$.

By conducting an inverse accumulated generating operation (IAGO), the predicated values are obtained

(7) $${\hat{x}}_{1}^{\left(0\right)}\left(k+1\right)={\hat{x}}_{1}^{\left(1\right)}\left(k+1\right)-{\hat{x}}_{1}^{\left(1\right)}\left(k\right)$$

### 3.3. Transformed Model of Nonlinear Grey Multivariable Models (TNGM (1, N))

**Definition** **4.** *The data series of the system sequence is*

$$X_{1}^{\left(0\right)}=\left(x_{1}^{\left(0\right)}(1),x_{1}^{\left(0\right)}(2),\cdots ,x_{1}^{\left(0\right)}(n)\right)$$

The data series of relevant factors are

$$\begin{array}{c} X_{2}^{\left(0\right)}=\left(x_{2}^{\left(0\right)}(1),x_{2}^{\left(0\right)}(2),\cdots ,x_{2}^{\left(0\right)}(n)\right)\\ X_{3}^{\left(0\right)}=\left(x_{3}^{\left(0\right)}(1),x_{3}^{\left(0\right)}(2),\cdots ,x_{3}^{\left(0\right)}(n)\right)\\ \vdots \\ X_{N}^{\left(0\right)}=\left(x_{N}^{\left(0\right)}(1),x_{N}^{\left(0\right)}(2),\cdots ,x_{N}^{\left(0\right)}(n)\right) \end{array}$$

$Z_{1}^{\left(1\right)}$ represents the data series of the nearest-neighbor mean of $X_{1}^{\left(1\right)}$ and is expressed as

(8) $$x_{1}^{\left(0\right)}(k)+az_{1}^{\left(1\right)}(k)=\sum_{i=2}^{N}b_{i}{\left(x_{i}^{\left(1\right)}(k)\right)}^{\gamma_{i}}$$

which is a nonlinear grey multivariable model, abbreviated as NGM (1, *N*) [52].

In the aforementioned model, $\gamma_{i}$ is the exponent of the i correlative variable, which can reflect the nonlinear influence of the i correlative variable on the variables of system behaviors. When $\gamma_{i}=1,(i=2,3,\cdots ,N)$, NGM (1, *N*) is the GM (1, *N*) model.

In real application, the basic conditions of constructing NGM (1, *N*) agree with the traditional GM (1, *N*) model in the research [49], namely, the 1-AGO for the raw data of the system behavior series has to follow grey exponential law, which can be satisfied in most of cases. Compared to the single variable grey predication model, the GM (1, *N*) model is more likely to show a drift of the data matrix in the recognition process of the parameters. Hence, it is better to preprocess raw data series with a large sample size before building the NGM (1, *N*) model. Concretely, data transformations based on initialization or mean quantization can be performed.

**Definition** **5.** *In the NGM (1, N) model,* −a *is the coefficient of system development,* $b_{i}{\left(x_{i}^{\left(1\right)}(k)\right)}^{\gamma_{i}}$ *denotes the driving item,* $b_{i}$ *is the driving factor coefficient, while* $\hat{a}={\left[a,b_{2},b_{3},\cdots ,b_{N}\right]}^{T}$ *is the data series of parameters.*

**Theorem** **3.** *Assuming that the data series of the system feature of* $X_{1}^{\left(0\right)}$*,* $X_{i}^{\left(0\right)}$(i=2,3,⋯,N) *is the data series of correlative factors,* $X_{i}^{\left(1\right)}$ *is the 1-AGO series of each* $X_{i}^{\left(0\right)}$ *value, and* $Z_{1}^{\left(1\right)}$ *is the data series of the nearest-neighbor mean of* $X_{1}^{\left(1\right)}$*.*

$$B=\left[\begin{array}{llll} -z_{1}^{\left(1\right)}(2) & {\left(x_{2}^{\left(1\right)}(2)\right)}^{\gamma_{2}} & \cdots & {\left(x_{N}^{\left(1\right)}(2)\right)}^{\gamma_{N}}\\ -z_{1}^{\left(1\right)}(3) & {\left(x_{2}^{\left(1\right)}(3)\right)}^{\gamma_{2}} & \cdots & {\left(x_{N}^{\left(1\right)}(3)\right)}^{\gamma_{N}}\\ \vdots & \vdots & \vdots & \vdots \\ -z_{1}^{\left(1\right)}(n) & {\left(x_{2}^{\left(1\right)}(n)\right)}^{\gamma_{2}} & \cdots & {\left(x_{N}^{\left(1\right)}(n)\right)}^{\gamma_{N}} \end{array}\right],Y=\left[\begin{array}{c} x_{1}^{\left(0\right)}(2)\\ x_{1}^{\left(0\right)}(3)\\ \vdots \\ x_{1}^{\left(0\right)}(n) \end{array}\right]$$

The estimates of the LSM for the parameter series $\hat{a}={\left[a,b_{2},b_{3},\cdots ,b_{N}\right]}^{T}$ satisfy the following conditions:

When n=N+1, $\hat{\alpha }=B^{-1}Y$ and |B|≠0;
When n>N+1, $\hat{\alpha }={\left(B^{T}B\right)}^{-1}B^{T}Y$ and $\left|B^{T}B\right|\neq 0$;
When n<N+1, $\hat{\alpha }=B^{T}{\left(B^{T}B\right)}^{-1}Y$ and $\left|B^{T}B\right|\neq 0$.

**Proof.**  by substituting k=2,3⋯,n into the NGM (1, *N*) model, $x_{1}^{\left(0\right)}(k)+az_{1}^{\left(1\right)}(k)=\sum_{i=2}^{N}b_{i}{\left(x_{i}^{\left(1\right)}(k)\right)}^{\gamma_{i}}$, we obtain the following equations:

(9) $$\begin{array}{c} x_{1}^{\left(0\right)}(2)=-az_{1}^{\left(1\right)}(2)+\sum_{i=2}^{N}b_{i}{\left(x_{i}^{\left(1\right)}(2)\right)}^{\gamma_{i}};\\ x_{1}^{\left(0\right)}(3)=-az_{1}^{\left(1\right)}(3)+\sum_{i=2}^{N}b_{i}{\left(x_{i}^{\left(1\right)}(3)\right)}^{\gamma_{i}};\\ \vdots \\ x_{1}^{\left(0\right)}(n)=-az_{1}^{\left(1\right)}(n)+\sum_{i=2}^{N}b_{i}{\left(x_{i}^{\left(1\right)}(n)\right)}^{\gamma_{i}}. \end{array}$$

The matrix form is written as:

(10) $$Y=B\hat{a}$$

When n=N+1, and |B|≠0, $\hat{\alpha }=B^{-1}Y$;
When n>N+1 and $\left|B^{T}B\right|\neq 0$, $\hat{\alpha }={\left(B^{T}B\right)}^{-1}B^{T}Y$;
When n<N+1, and B is a row-full-rank matrix, the full-rank decomposition of B is
  (11) B=DC
  the generalized inverse matrix $B^{+}$ of B is
  (12) $$B^{+}=C^{T}{\left(CC^{T}\right)}^{-1}{\left(DD^{T}\right)}^{-1}D^{T}$$
  (13) $$\hat{\alpha }=C^{T}{\left(CC^{T}\right)}^{-1}{\left(DD^{T}\right)}^{-1}D^{T}Y$$
  by taking D as the identity matrix $I_{n-1}$, we obtain $\hat{\alpha }=B^{T}{\left(B^{T}B\right)}^{-1}Y$.

**Definition** **6.** *Assuming that* $\hat{a}={\left[a,b_{2},b_{3},\cdots ,b_{N}\right]}^{T}$*,*

(14) $$\frac{dx_{1}^{\left(1\right)}(t)}{dt}+ax_{1}^{\left(1\right)}(t)=\sum_{i=2}^{N}b_{i}{\left(x_{i}^{\left(1\right)}(t)\right)}^{\gamma_{i}}$$

*is obtained which is the whitening differential equation of the NGM (1, N) model.*

**Theorem** **4.** *Supposing that* $X_{i}^{\left(0\right)}$*,* $X_{i}^{\left(1\right)}\left(2,3,\ldots ,N\right)$ *and* $Z_{1}^{\left(1\right)}$ *are presented as* B,Y *Theorem 1, and the solution of the whitening differential equation* $\frac{dx_{1}^{\left(1\right)}(t)}{dt}+ax_{1}^{\left(1\right)}(t)=\sum_{i=2}^{N}b_{i}{\left(x_{i}^{\left(1\right)}(t)\right)}^{\gamma_{i}}$ *is*

(15) $$\begin{array}{cc} x_{1}^{\left(1\right)}(t) & =e^{-at}\left\{\sum_{i=2}^{N}\int b_{i}{\left(x_{i}^{\left(1\right)}(t)\right)}^{\gamma_{i}}e^{at}dt+x_{{}^{1}}^{\left(1\right)}(0)-\sum_{i=2}^{N}\int b_{i}{\left(x_{i}^{\left(1\right)}(0)\right)}^{\gamma_{i}}dt\right\}\\ & =e^{-at}\left\{x_{{}^{1}}^{\left(1\right)}(0)-t\sum_{i=2}^{N}b_{i}{\left(x_{i}^{\left(1\right)}(0)\right)}^{\gamma_{i}}+\sum_{i=2}^{N}\int b_{i}{\left(x_{i}^{\left(1\right)}(t)\right)}^{\gamma_{i}}e^{at}dt\right\} \end{array}$$

$\sum_{i=2}^{N}b_{i}{\left(x_{i}^{\left(1\right)}(k)\right)}^{\gamma_{i}}$ *can be seen as a grey constant, when* $X_{i}^{\left(1\right)}\left(i=2,3,\ldots ,N\right)$ *changes slightly, while the approximate time response of NGM (1, N) is expressed as*

(16) $${\hat{x}}_{1}^{\left(1\right)}(k+1)=\left[x_{1}^{\left(1\right)}(1)-\frac{1}{a}\sum_{i=2}^{N}b_{i}{\left(x_{i}^{\left(1\right)}(k+1)\right)}^{\gamma_{i}}\right]e^{-ak}+\frac{1}{a}\sum_{i=2}^{N}b_{i}{\left(x_{i}^{\left(1\right)}(k+1)\right)}^{\gamma_{i}}$$

By conducting IAGO, the predicated values are obtained

(17) $${\hat{x}}_{1}^{\left(0\right)}(k+1)={\hat{x}}_{1}^{\left(1\right)}(k+1)-{\hat{x}}_{1}^{\left(1\right)}(k)$$

which is the approximate solution of the time response function for the whitening differential equation in the aforementioned NGM (1, *N*) model. In real application, $X_{i}^{\left(1\right)}\left(i=2,3,\ldots ,N\right)$ may present a great change, $\sum_{i=2}^{N}b_{i}{\left(x_{i}^{\left(1\right)}(k)\right)}^{\gamma_{i}}$; therefore, it cannot be seen as a grey constant. Hence, Equation (16) is applicable to the real case, as it may cause a large error. To address this problem, a derived model based on NGM (1, *N*) is used to replace [52]. Equation (16) is applied in simulation and predication.

**Theorem** **5.** The derived model based on the definition of NGM (1, N) is deduced and abbreviated as TNGM (1, N):

(18) $$x_{1}^{\left(0\right)}(k)=\sum_{i=2}^{N}\beta_{i}{\left(x_{i}^{\left(1\right)}(k)\right)}^{\gamma_{i}}-\alpha x_{1}^{\left(1\right)}(k-1)$$

*where* $\beta_{i}=\frac{b_{i}}{1+0.5a}$*,* $\alpha =\frac{a}{1+0.5a}$*.*

**Proof.**  Due to the background value of $x_{1}^{\left(0\right)}(k)$, it is obtained as

(19) $$\begin{array}{c} z_{1}^{\left(1\right)}(k)=0.5x_{1}^{\left(1\right)}(k-1)+0.5x_{1}^{\left(1\right)}(k-1)+0.5x_{1}^{\left(0\right)}(k)\\ =x_{1}^{\left(1\right)}(k-1)+0.5x_{1}^{\left(0\right)}(k) \end{array}$$

by substituting Equation (11) into the defined NGM (1, *N*), we obtain

(20) $$x_{1}^{\left(0\right)}(k)+a\left[x_{1}^{\left(1\right)}(k-1)+0.5x_{1}^{\left(0\right)}(k)\right]=\sum_{i=2}^{N}b_{i}{\left(x_{i}^{\left(1\right)}(k)\right)}^{\gamma_{i}}$$

that is

(21) $$\left(1+0.5a\right)x_{1}^{\left(0\right)}(k)=\sum_{i=2}^{N}b_{i}{\left(x_{i}^{\left(1\right)}(k)\right)}^{\gamma_{i}}-ax_{1}^{\left(1\right)}(k-1)$$

the solution is

(22) $$x_{1}^{\left(0\right)}(k)=\sum_{i=2}^{N}\frac{b_{i}}{1+0.5a}{\left(x_{i}^{\left(1\right)}(k)\right)}^{\gamma_{i}}-\frac{a}{1+0.5a}x_{1}^{\left(1\right)}(k-1)$$

In the case of $\gamma_{i}=1,(i=2,3,\cdots ,N)$, TNGM (1, *N*) is degenerated to be a derived model of GM (1, *N*) in the research [49].

### 3.4. Parameter Estimation of the Transformed Model of the Nonlinear Grey Multivariable Model Based on NLS(NLS-TNGM (1, N))

According to the aforementioned deduction process, Equation (8) presents a nonlinear relationship between the grey derivative and unknown parameters. The first-order condition of the nonlinear model remains, considered as the nonlinear function of estimate parameters, so the conventional LSM does not fit for solving the parameters in this equation; they need to be solved using a complex optimized algorithm. NLS is a parameter estimation approach that uses square error and minimum error as benchmarks to estimate the parameters of a nonlinear static model [53]. It is used to fit a group of m measuring values, which involves the nonlinear model containing unknown parameters (*m* > *n*). This approach approximates the solution of the model using a linear method. By continuous iteration to extract parameters, it is able to continuously approximate the optimal regression coefficient of the nonlinear regression model. Hence, the transformed nonlinear grey multivariable based on Nonlinear least squares method-(NLS-TNGM (1, *N*)) is proposed for estimation of the parameters by using the Gauss–Seidel iteration method as the iteration approach [54]. Each iteration of the Gauss–Seidel iterative algorithm makes full use of the current iteration value, that is, when the component is calculated, the newest components $x_{1}^{\left(k+1\right)}$, $x_{2}^{\left(k+1\right)}$, …, $x_{i-1}^{\left(k+1\right)}$, are used to replace the old components $x_{1}^{\left(k\right)}$, $x_{2}^{\left(k\right)}$, …, $x_{i-1}^{\left(k\right)}$, so as to save storage space and accelerate the iteration process. The main steps are described as follows:

**Step 1: Determination of the initial vector**

The conduction of the iteration estimation requires the initial values of the coefficients to be solved in the model. The parameters sequence to be estimated in TNGM (1, *N*) is $\hat{\alpha }={\left[a,b_{2},r_{2}\right]}^{T}$. As there is no general rule to determine the initial values of parameters, the initial values of the parameters sequence to be solved in this research are set as a=0, $b_{2}=0$ and $r_{2}=0$.

**Step 2: Calculation and estimation of parameters**

The parameter vectors of TNGM (1, *N*) can be estimated using Equation (8):

(23) $$x_{1}^{\left(0\right)}(k)=-az_{1}^{\left(1\right)}(k)+{\sum_{i=2}^{N}b_{i}\left(x_{i}^{\left(1\right)}\left(k\right)\right)}^{\gamma_{i}}\left(i=2,3,\ldots ,N\right)$$

Based on this equation, a new component $x_{1}^{\left(0\right)}\left(k\right)$ can be calculated, which is further calculated for a newly generated iterated value $x_{1}^{\left(0\right)}\left(k+1\right)$.

**Step 3: Iteration**

The parameters of Step 2 can be estimated using Equation (23). If the parameter vector obtained by the j iteration is $\alpha_{j}$, the corresponding linear model acquired by the (j+1) iteration is expressed as:

(24) $$\alpha_{j+1}=\alpha_{j}+\Delta_{j}$$

According to the same method, this process is repeated until the condition of the stopping iteration is satisfied.

**Step 4: The law of iteration stopping**

The stopping rule of the iteration calculation in this work is based on the change after each iteration of the parameters to be estimated—the deviation of the results for two neighboring iterations—and is calculated as

(25) $$e=\underset{1\leq i\leq n}{\max}\left|x_{i}^{\left(k+1\right)}-x_{i}^{\left(k\right)}\right|$$

**Figure 1:**
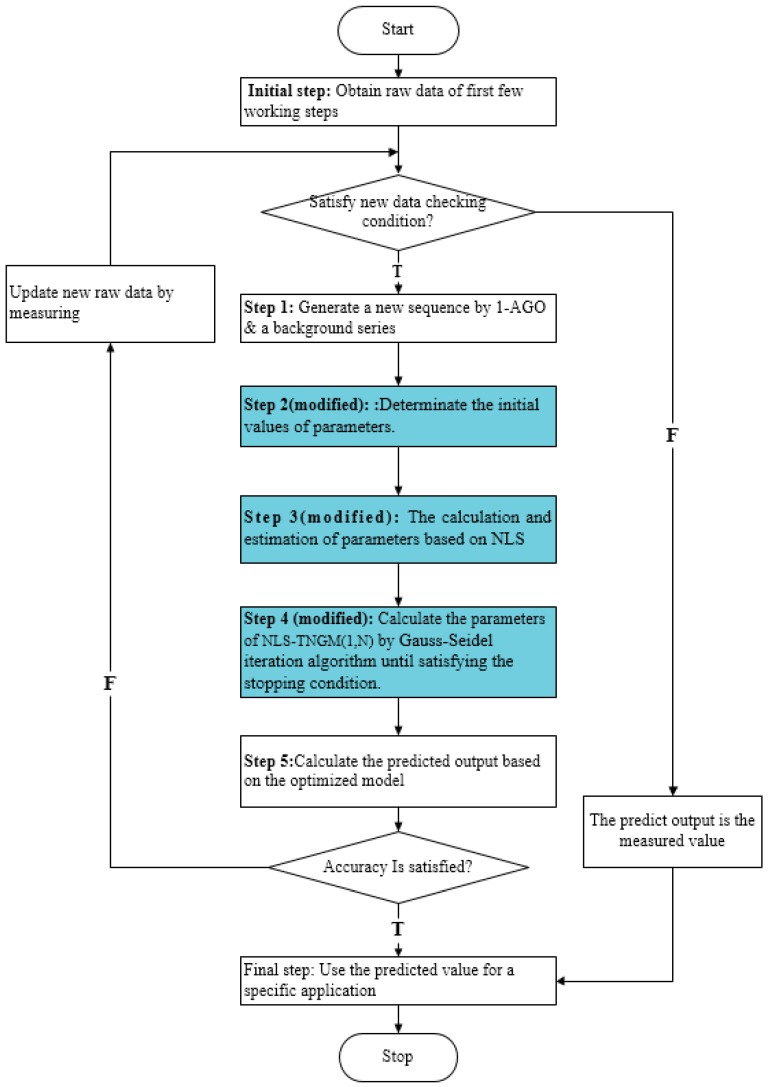
The calculation steps using the transformed nonlinear grey multivariable TNGM (1, *N*) based on Nonlinear least squares method.

When the maximum change of the parameters to be estimated is smaller than that of a preset value, the iteration stops: the iteration stops when the deviation e is below preset forecasting precision values. The above improved modeling process can be expressed by the flow chart shown in Figure 1.

## 4. Empirical Analysis

To verify the feasibility and accuracy of the predication models, the nonlinear model is constructed to predicate the nonlinear relationship between WDPC, SO_2_ and dust emissions per capita, and economic growth based on China’s domestic data. Furthermore, GM (1, *N*) and NLS-TNGM (1, *N*) models are built respectively to predicate the same data. The predicated results of three types of the model are compared and analyzed as follows.

### 4.1. The Explanation of Variables and Data

This research selects three pollutants (WDPC, SO_2_ and dust) during the period 1996–2015 to perform an empirical analysis. The data of GDP per capita, and the wastewater discharge per capita (WDPC), SO_2_ and dust emissions per capita are sourced from National Bureau of Statistics of China (http://www.stats.gov.cn/tjsj). To overcome the influence of inflation, real GDP per capita is obtained by processing GDP indexes and corresponding data are shown in Table 1.

### 4.2. The Establishment and Solution of the Predication Models

#### 4.2.1. GM (1, *N*) Model

Equations (1)–(7) are employed to build the GM (1, *N*) model based on three groups of data and corresponding parameters are displayed in Table 2.

Each initial value is set as $x_{1}^{\left(1\right)}\left(1\right)=1$; the equations of the corresponding time response can be written as

WDPC: ${\overset{⌢}{x}}_{1}^{\left(1\right)}\left(k+1\right)=\left(1-0.231\times x_{2}^{\left(1\right)}\left(k+1\right)\right)\times e^{0.1971\times k}+0.231\times x_{2}^{\left(1\right)}\left(k+1\right)$
SO_2_ emissions per capita: ${\overset{⌢}{x}}_{1}^{\left(1\right)}\left(k+1\right)=\left(1-0.228\times x_{2}^{\left(1\right)}\left(k+1\right)\right)\times e^{0.1871\times k}+0.228\times x_{2}^{\left(1\right)}\left(k+1\right)$
Dust emissions per capita: ${\overset{⌢}{x}}_{1}^{\left(1\right)}\left(k+1\right)=\left(1-0.2201\times x_{2}^{\left(1\right)}\left(k+1\right)\right)\times e^{0.1407\times k}+0.2201\times x_{2}^{\left(1\right)}\left(k+1\right)$

The predicated and actual values obtained based on the aforementioned time response equations are shown in Table 3.

#### 4.2.2. The NLS-TNGM (1, *N*) Model

Based on three groups of data, the NLS-TNGM (1, *N*) model is built and solved using Eviews8 software. The corresponding parameters of the model are shown in Table 4.

Each initial value is set as $x_{1}^{\left(1\right)}\left(1\right)=1$, and the equations of the corresponding time response are expressed as

WDPC: ${\overset{⌢}{x}}_{1}^{\left(1\right)}\left(k+1\right)=\left(1+23.85\times {\left(x_{2}^{\left(1\right)}\left(k+1\right)\right)}^{-0.099}\right)\times e^{0.0426\times k}-23.85\times {\left(x_{2}^{\left(1\right)}\left(k+1\right)\right)}^{-0.099}$
SO_2_ emissions per capita: ${\overset{⌢}{x}}_{1}^{\left(1\right)}\left(k+1\right)=\left(1-57.86\times {\left(x_{2}^{\left(1\right)}\left(k+1\right)\right)}^{0.06658}\right)\times e^{-0.0172\times k}+57.86\times {\left(x_{2}^{\left(1\right)}\left(k+1\right)\right)}^{0.06658}$
Dust emissions per capita: ${\overset{⌢}{x}}_{1}^{\left(1\right)}\left(k+1\right)=\left(1+52.889\times {\left(x_{2}^{\left(1\right)}\left(k+1\right)\right)}^{-0.5574}\right)\times e^{0.0525\times k}-52.889\times {\left(x_{2}^{\left(1\right)}\left(k+1\right)\right)}^{-0.5574}$

The results compared between the actual values and predicated values based on the time response equations are shown in Table 3.

### 4.3. Comparison of Modeling Results

**Figure 2:**
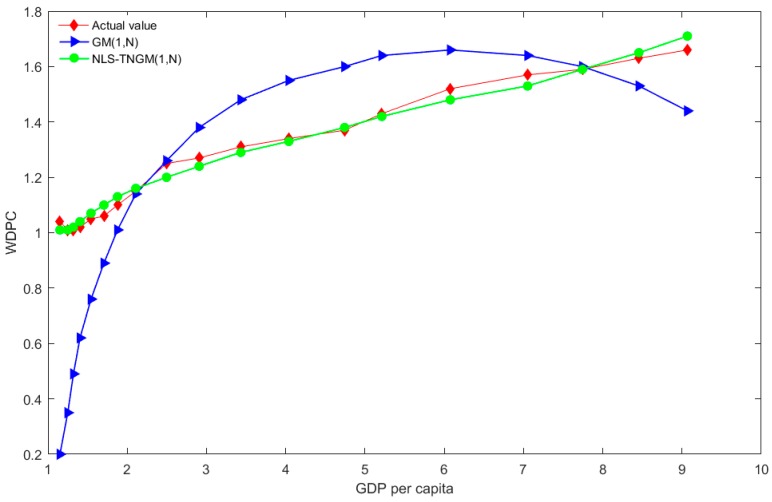
The distributions of forecasted WDPC values using the two models, and actual values.

**Figure 3:**
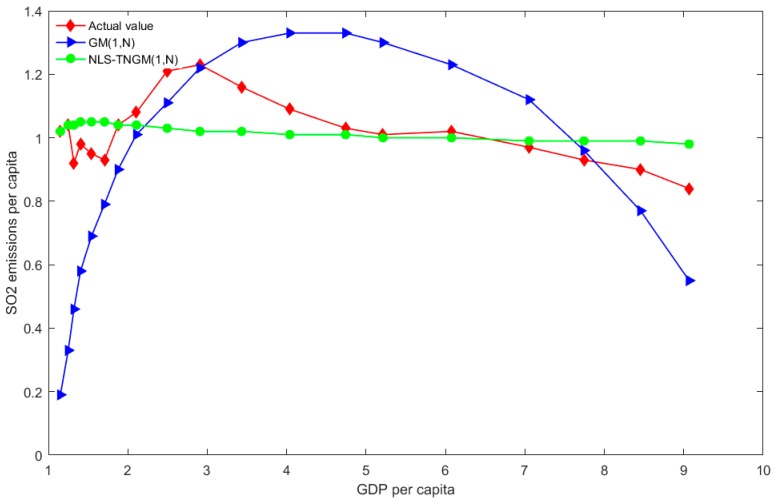
The distributions of forecasted values of SO_2_ emissions per capita using the two models, and actual values.

The compared results of the actual values and predicated values using the aforementioned GM (1, *N*) and NLS-TNGM (1, *N*) models are displayed in Table 4. For the sake of comparison, four groups of predicated and actual values are illustrated in Figure 1, Figure 2 and Figure 3. 

Table 4 displays the simulated and forecasted data concerning WDPC, and the per capita emissions of SO_2_ and dust using the GM (1, *N*) and NLS-TNGM (1, *N*) models. As shown in Table 4, the forecasted mean absolute percent errors (MAPEs) of WDPC, and the per capita emissions of SO_2_ and dust using NLS-TNGM (1, *N*) are 1.72%, 7.33% and 11.06% respectively, while the forecasted MAPEs for those using GM (1, *N*) are 20.8%, 25.77% and 32.36% respectively. The results indicated that NLS-TNGM (1, *N*) presents greater forecasting precision compared to GM (1, *N*). As illustrated in Figure 2, the forecasted results of WDPC using GM (1, *N*) do not agree with actual values. As seen from the figure, the actual value of WDPC is shown to be a monotonously increasing curve, while the fitting curve of GM (1, *N*) is characterized by an initial increase followed by a decrease. In contrast, the fitting curves of NLS-TNGM (1, *N*) generally agree with actual values, and the prediction results of the model are in line with the assumptions in the previous section; there is a monotonically increasing relationship between GDP per capita and WDPC.

As shown in Figure 3, the curve of forecasted per capita SO_2_ emissions based on GM (1, *N)* presents an inverted U shape; however, the curve of actual per capita SO_2_ emissions is shown to fluctuate slightly and then gradually decrease. The curve of the fitted values of NLS-TNGM (1, *N*) is smooth. Although the fitted values are inconsistent with the shapes of actual values, the forecasting precision of NLS-TNGM (1, *N*) is far better than that of the GM (1, *N*) model by integrating the MAPEs predicated based on NLS-TNGM (1, *N*). This is because the fitted value ranges between 0.9–1.1, showing a small fluctuation, which is therefore unobvious in the curve. Overall, the model predictions agree well with the assumptions in the previous section; there is a decreasing relationship between SO_2_ emissions per capita and GDP per capita.

**Figure 4:**
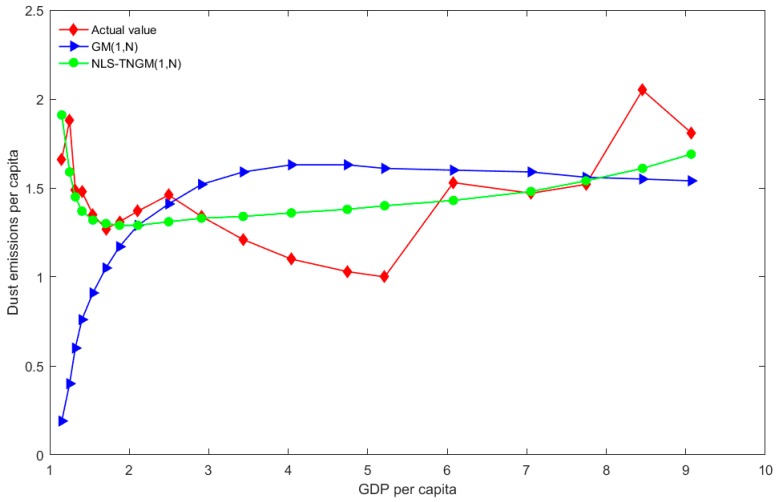
The distributions of forecasted values of dust emissions per capita using the two models, and actual values.

As illustrated in Figure 4, forecasted per capita SO_2_ emissions using GM (1, *N*) initially increase and then tend to be steady, while the actual values fluctuate violently. In general, the actual values initially decline and then increase; the curve of fitted values of NLS-TNGM (1, *N*) presents the same change, which is more in agreement with the nonlinear feature of actual values. The predicted values of the model are consistent with the above assumptions; there is a relationship between dust emissions per capita and per capita GDP, i.e., initially decreasing and then increasing.

Comprehensive empirical results show that WDPC indicates a growing tendency aligned with the growth of GDP, while the per capita emissions of SO_2_ and dust reduce accordingly. In the Fifth Plenary Session of the 18th CPC Central Committee, green development is ranked as one of the five development concepts during the 13th Five-Year Plan period: the goals to achieve overall eco-environmental sustainability and largely reduce the total pollutant discharges are stipulated. Pollutant discharge has been an important measure and represents a breakthrough for controlling the eco-environment. Nowadays, China is experiencing a key stage in its transformation and development; therefore, environmental governance has been a major focus of the Chinese government and has witnessed some achievements. To further promote the green development concept, the Chinese government has suggested the implementation of corresponding policy to achieve pollutant reductions according to real cases. Furthermore, the Chinese government also needs to comprehensively accelerate the zero-pollutant discharge and pollutant reduction, as well as slow down the degree of environmental pollution while ensuring the sustainable growth of economic development.

## 5. Conclusions

Considering that the conventional LSM is applicable to solving nonlinear issues, the Gauss–Seidel iteration algorithm is used to propose a new approach for solving the parameters of TNGM (1, *N*) based on the basic principle of NLS. By conducting continuous iteration to constantly approximate the optimal regression coefficient of the nonlinear model, the forecasted precision of the model can be improved. WDPC and the per capita emissions of SO_2_ and dust are empirically analyzed and the results are compared with the traditional GM (1, *N*) model, so as to verify the effectiveness and superiority of the proposed new model. The model’s predictions for the emissions of three pollutants are in line with the previous assumptions. There is a monotonically increasing relationship between GDP per capita and WDPC, and a decreasing relationship between SO_2_ emissions per capita and GDP per capita, and a relationship between dust emissions per capita and per capita GDP which initially decreases and then increases. NLS-TNGM (1, *N*) is able to effectively recognize the parameters of TNGM (1, *N*), which can be applied in the research of nonlinear time series of a small sample size. However, the method has limitations in the predication of nonlinear time series of a large sample size. The applicability of this model warrants further analysis. This is mainly because a large sample series is likely to generate more turning points, and the form of exponents is not favorably applicable in the fluctuating data series. In the context of solving parameters based on NLS, TNGM (1, *N*) needs to be further comprehensively analyzed, including the modeling method of background values, initial conditions and structural variables.

## Figures and Tables

**Table 1:** The actual value of GDP per capita and emissions of three pollutants from 1996 to 2015.

Year	GDP per Capita (Yuan)	Wastewater Discharge per Capita—WDPC (Ton)	SO_2_ Emissions per Capita (Ton)	Dust Emissions per Capita (Ton)
1996	5539.01	32.2193	0.0161	0.0062
1997	6375.74	33.6343	0.0164	0.0103
1998	6921.71	32.4432	0.0168	0.0117
1999	7319.62	32.5936	0.0148	0.0092
2000	7778.40	32.7592	0.0157	0.0092
2001	8545.59	33.9270	0.0153	0.0084
2002	9449.23	34.2148	0.0150	0.0079
2003	10,399.56	35.5421	0.0167	0.0081
2004	11,679.27	37.1111	0.0173	0.0085
2005	13,823.11	40.1511	0.0195	0.0090
2006	16,106.53	40.8527	0.0197	0.0083
2007	19,014.37	42.1558	0.0187	0.0075
2008	22,370.96	43.0716	0.0175	0.0068
2009	26,267.77	44.1439	0.0166	0.0064
2010	28,870.42	46.0359	0.0163	0.0062
2011	33,654.84	48.9251	0.0165	0.0095
2012	39,060.42	50.5717	0.0156	0.0091
2013	42,887.50	51.1085	0.0150	0.0094
2014	46,833.94	52.3589	0.0144	0.0127
2015	50,223.99	53.4928	0.0135	0.0112

Note: As China’s wastewater discharge data in 1996, 1998 and 1999 and the SO_2_ per capita emissions data in 1994, 1996 and 1998 are absent, they were obtained using the mean substitution method. To eliminate issues regarding different dimensional variables, the authors initialized the raw data in Section 4.

**Table 2:** The coefficients of GM (1, *N*) for three pollutants.

Coefficients	WDPC	SO_2_ Emissions per Capita	Dust Emissions per Capita
a	−0.1971	−0.18712	−0.14073
$b_{2}$	−0.04553	−0.04268	−0.03109

**Table 3:** Forecasting values and errors of three pollutants using two models.

**WDPC**						**SO_2_ Emissions per Capita**					
**Year**	**Actual Value**	**GM (1, *N*)**		**NLS-TNGM (1, *N*)**		**Year**	**Actual Value**	**GM (1, *N*)**		**NLS-TNGM (1, *N*)**	
		**Model Value**	**Error**	**Model Value**	**Error**			**Model Value**	**Error**	**Model Value**	**Error**
1996	1.00	1.00	0.00	1.00	0.00	1996	1.00	1.00	0.00	1.00	0.00
1997	1.04	0.20	80.65	1.01	3.49	1997	1.02	0.19	81.31	1.02	−0.05
1998	1.01	0.35	65.51	1.01	−0.24	1998	1.04	0.33	68.29	1.04	0.62
1999	1.01	0.49	51.96	1.02	−1.19	1999	0.92	0.46	50.17	1.04	−13.59
2000	1.02	0.62	38.83	1.04	−2.74	2000	0.98	0.58	41.25	1.05	−6.83
2001	1.05	0.76	28.23	1.07	−1.62	2001	0.95	0.69	27.34	1.05	−10.21
2002	1.06	0.89	16.52	1.10	−3.47	2002	0.93	0.79	14.99	1.05	−12.04
2003	1.10	1.01	8.05	1.13	−2.47	2003	1.04	0.90	13.62	1.04	−0.33
2004	1.15	1.14	0.97	1.16	−1.14	2004	1.08	1.01	6.78	1.04	3.82
2005	1.25	1.26	−1.37	1.20	3.48	2005	1.21	1.11	8.16	1.03	14.93
2006	1.27	1.38	−8.73	1.24	1.92	2006	1.23	1.22	0.59	1.02	16.40
2007	1.31	1.48	−12.83	1.29	1.71	2007	1.16	1.30	−11.44	1.02	12.44
2008	1.34	1.55	−16.18	1.33	0.50	2008	1.09	1.33	−22.59	1.01	6.95
2009	1.37	1.60	−17.07	1.38	−0.42	2009	1.03	1.33	−28.75	1.01	2.42
2010	1.43	1.64	−14.95	1.42	0.31	2010	1.01	1.30	−28.05	1.00	1.09
2011	1.52	1.66	−9.08	1.48	2.77	2011	1.02	1.23	−20.06	1.00	2.51
2012	1.57	1.64	−4.46	1.53	2.42	2012	0.97	1.12	−14.65	0.99	−2.20
2013	1.59	1.60	−0.74	1.59	−0.17	2013	0.93	0.96	−3.12	0.99	−6.00
2014	1.63	1.53	5.88	1.65	−1.42	2014	0.90	0.77	13.76	0.99	−9.91
2015	1.66	1.44	13.24	1.71	−2.97	2015	0.84	0.55	34.62	0.98	−16.86
MAPE			20.80		1.72				25.77		7.33
**Dust Emissions per Capita**						**Dust Emissions per Capita**					
**Year**	**Actual Value**	**GM (1, *N*)**		**NLS-TNGM (1, *N*)**		**Year**	**Actual Value**	**GM (1, *N*)**		**NLS-TNGM (1, *N*)**	
		**Model Value**	**Error**	**Model Value**	**Error**			**Model Value**	**Error**	**Model Value**	**Error**
1996	1.00	1.00	0.00	1.00	0.00	2006	1.34	1.52	−13.69	1.33	0.84
1997	1.66	0.19	88.52	1.91	−14.74	2007	1.21	1.59	−32.08	1.34	−11.50
1998	1.88	0.40	78.68	1.59	15.43	2008	1.10	1.63	−48.59	1.36	−24.14
1999	1.49	0.60	59.83	1.45	2.78	2009	1.03	1.63	−59.01	1.38	−34.24
2000	1.48	0.76	48.60	1.37	7.96	2010	1.00	1.61	−61.44	1.40	−39.87
2001	1.35	0.91	32.42	1.32	2.34	2011	1.53	1.60	−4.47	1.43	6.57
2002	1.27	1.05	17.80	1.30	−1.82	2012	1.47	1.59	−8.12	1.48	−0.59
2003	1.31	1.17	10.72	1.29	1.79	2013	1.52	1.56	−3.05	1.54	−1.28
2004	1.37	1.29	5.37	1.29	5.46	2014	2.05	1.55	24.50	1.61	21.73
2005	1.46	1.41	3.19	1.31	10.58	2015	1.81	1.54	14.69	1.69	6.41
MAPE									32.36		11.06

$Error=\frac{x_{1}^{\left(0\right)}\left(k\right)-{\hat{x}}_{1}^{\left(0\right)}\left(k\right)}{x_{1}^{\left(0\right)}\left(k\right)}\times 100\%$.

**Table 4:** The coefficients of NLS-TNGM (1, *N*) for three pollutants.

Coefficients	WDPC	SO_2_ Emissions per Capita	Dust Emissions per Capita
a	−0.042633	0.017207	−0.052495
$b_{2}$	1.016804	0.995608	2.776413
$r_{2}$	−0.098991	0.06658	−0.557439
